# From microbial technology to microbiota medicine as a clinical discipline: Sustainable development goal

**DOI:** 10.1111/1751-7915.14317

**Published:** 2023-07-15

**Authors:** Faming Zhang, Weihong Wang, Yongzhan Nie, Jingnan Li, Xingxiang He

**Affiliations:** ^1^ Department of Microbiota Medicine & Medical Center for Digestive Diseases The Second Affiliated Hospital of Nanjing Medical University Nanjing China; ^2^ State Key Laboratory of Cancer Biology and Xijing Hospital of Digestive Diseases Xijing Hospital, Air Force Medical University Xi'an China; ^3^ Department of Gastroenterology, Peking Union Medical College Hospital Chinese Academy of Medical Sciences and Peking Union Medical College Beijing China; ^4^ Department of Gastroenterology The First Affiliated Hospital of Guangdong Pharmaceutical University Guangzhou China

## MICROBIOME‐BASED THERAPEUTICS: BREAKTHROUGH, WORRY AND ACTION

Health and well‐being are important components of the United Nations Sustainable Development Goals. Research on microbiota and human health is a burning question in achieving these goals. Global use of antibiotics and other drugs, the prevalence of the ultra‐processed foods in the Western diet, drastic changes in lifestyle and industrial development continue to damage both environmental and human microbiota (Marchesi et al., 2016). Numerous studies have shown that human microbiota plays an important role in disease profiles, from infections, inflammation, malnutrition to cancer, providing a new dimension for understanding medicine and life science.

The recognition of the importance of the microbiome in health and disease, particularly in inflammation and immune function, has become one of the most significant scientific breakthroughs in the past decade. Targeting the human microbiome for diagnosis and treatment of diseases has led to a series of groundbreaking technologies, especially in microbiome sequencing, multiomics integration, and faecal microbiota transplantation (FMT), which are changing clinical practice. From the perspective of using microbial cells to treat diseases, probiotics and FMT are at two extremes. FMT being the most effective clinical technology fundamentally proves the importance of the microbiome in human health and disease. An analysis of global FMT clinical reports in the 10 years since the term ‘fecal microbiota transplantation’ was defined in 2011, FMT has been used to treat 85 diseases related to microbiome dysbiosis, which can be collectively referred to as microbiota dysbiosis‐related diseases (Wang et al., 2022). These diseases are related to dysbiosis and can be treated by microbiome reconstruction. In recent years, new technologies derived from FMT have emerged, mainly including washed microbiota transplantation (WMT) based on medical devices approved in China (Lu et al., 2022; Wang et al., 2022), faecal liquid enemas as drug approved in the United States (Khanna et al., 2022) and purified spores from faeces as drug approved in the United States (Feuerstadt et al., 2022). This rapidly increasing clinical evidence has broken through the boundaries of classical digestive system diseases, highlighting the transformative role of microbiome‐targeted technologies.

Developing microbiome‐based therapeutics is the driving force moving clinical medicine forward. How can we maximize the use of human microbiome to diagnose and treat individual diseases and promote human health? In fact, the number of patients who actually benefited from emerging microbiome technologies was far from the theoretical number of beneficiaries. If these microbiome technologies are allowed to develop freely according to the current form and promoted and developed according to the rules of technical commercialization, the entire field will face bottleneck constraints. The use of technical thinking, utilitarian behaviour and commercial activities may result in medical unfairness due to high costs. Additionally, excessive reliance on technology can lead to ethical and moral issues, which can create obstacles for the development of the field. To solve these bottleneck problems and avoid these potential traps, a scientific framework higher than the technology itself is needed to achieve sustainable development goals. However, in the existing clinical medicine discipline classification system, there is no discipline that can cover the above medical needs.

Solving the emerging microbiome‐based clinical medical problems is an important action to transform our world and achieve sustainable development goals. After Faming Zhang received the invitation from Juan Luis Ramos, the editor of *Microbial Biotechnology* in 2023 for contributing an article which highlights the potential contributions of microbes to achieving sustainable development goals in clinical medicine, the authors of this article prepared for this and held a dialogue and discussion on the theme of ‘From Microbial Technology to Microbiota Medicine: Sustainable Development Goals’ at the China Gut Conference (China National Convention Center, Beijing) on May 21, 2023 and then revised and formed this article.

## HOW TO UNDERSTAND THE DEFINITION OF MICROBIOTA MEDICINE?

The human microbiota has been considered as an organ, an intrinsic feature of human developmental environment, or integrated into the human body as a superorganism, and these understandings have been the focus of scientific debate. From the perspective of microbiology, microbiota should be expressed as microbial communities; from the perspective of holistic integrative medicine, microbiota should be understood as a superorganism. Although the term microbiome has multiple meanings, it essentially covers the molecular research projects of ecology or microecology in the genomic era, and similar ‘‐ome’ terms include virome, genome, transcriptome and proteome. Microecology is the study of the mechanisms and laws of the interaction between microorganisms and their hosts (including plants, animals and humans) and belongs to the field of ecology (Huss, 2014). Microecology was mainly used in the scientific context of the 1960s to 1980s. In the past 20 years, research on microecology has developed rapidly, and the biomedical field mainly uses ‘microbiota’ or ‘microbiome’.

There are many potential techniques used for diagnosing and treating human diseases after years of collective exploration worldwide. Microbiota medicine has been born as a clinical medicine discipline since 2023 in Nanjing Medical University, China (Zhang, 2023). The definition of microbiota medicine (or microecological medicine, microbiota/microbiome‐based medicine) is the basic theory and diagnostic and therapeutic techniques for studying the interaction between microbial communities and the human host, diagnosis, treatment and disease prevention and clinical medicine education, which belongs to the branch of clinical medicine.

Microbiota medicine is different from medical microbiology (or medical microecology), and this should not be a confused issue, just as anatomy and surgery have essential differences in their tasks. Medical microbiology belongs to the category of basic medicine, and its core task is rational medicine; microbiota medicine belongs to the category of clinical medicine, and its core task is effective diagnosis and treatment.

Microbiota medicine satisfies the conditions required to become a discipline, including (1) a specific conceptual system that supports the formation and development of microbiota medicine concepts, such as microbiology, metagenomics and metabolomics, which have been widely used in life sciences research; (2) a network of relationships between these concepts that serve clinical needs, allowing for the understanding and formation of a specific logical structure for microbiota‐based prevention, diagnosis and treatment, such as the development from the Human Microbiome Project to the Integrated Human Microbiome Project (Integrative HMP (iHMP) Research Network Consortium, 2014), as well as the gut–brain and gut–liver axes; (3) microbiota‐based knowledge statements that can be verified by clinical experience, such as the continued use of FMT, the significant changes in the human disease spectrum in recent decades, and health issues in the postantibiotic era; and (4) the development of specific technologies for explaining, teaching and clinically applying microbiota medicine, such as WMT (Fecal Microbiota Transplantation‐standardization Study Group, 2020), repeated microbiota transplantation via colonic transendoscopic enteral tubing (TET) (Wang et al., 2023), spore‐based pharmaceutical technology (Feuerstadt et al., 2022), phage combination therapy (Federici et al., 2022) and specific dietary fibre treatments (Han et al., 2022; Zhao et al., 2018).

Microbiota is closely related to health and disease, involving the entire human life cycle and all organs of the body, spanning multiple disciplines and incorporating various technologies. Microbiota medicine is a new clinical medicine discipline that is suitable for independent establishment in teaching hospitals of universities with the necessary conditions. It should be positioned as a secondary discipline under clinical medicine, encouraging multidisciplinary talents with interest, passion and professional skills to form new discipline teams to carry out scientific research, practice the discipline framework, develop guidelines, establish national technical standards and cultivate talents for microbiota medicine. Therefore, one of the important goals of microbiota medicine is to cultivate professional and research‐oriented medical personnel for the prevention, diagnosis and treatment of human microbiota‐related diseases. The educational process of microbiota medicine will become an important means of integrating interdisciplinary education. Compared with traditional clinical medicine disciplines, professionals in the field of microbiota medicine need a broader perspective, more skills and better ability to handle complex and critical illnesses. For example, patients with diabetic peripheral neuropathy and epilepsy usually seek treatment in endocrinology and neurology departments, respectively. These doctors need to have the correct decision‐making ability regarding microbiota transplantation and the delivering techniques required.

The subdisciplines of microbiota medicine include basic microbiota technology, clinical microbiota treatment technology and microbiota medicine management. The main task of basic microbiota technology is to carry out microbiota laboratory technology services, research and education around clinical needs, such as donor recruitment, microbiota preparation, component analysis, technical quality control, safety evaluation, microbiota diagnosis and engineering technology research. The main task of clinical microbiota treatment technology is to directly provide microbiota treatment services, research and education to patients, such as clinical decision‐making, endoscopic intervention technology for microbiota diagnosis and treatment, and microbiota communities treatment. The main task of microbiota medicine management is to provide services, research and education for microbiota medicine health policies, professional talents training, cost‐effectiveness and other aspects for patients.

## HOW TO UNDERSTAND THE DIMENSIONS OF MICROBIOTA MEDICINE?

Due to its outstanding interdisciplinary nature, microbiota medicine has multiple dimensions. Specific research and clinical practices in microbiota medicine include the study of the interaction between microbiome and the host, microbiome diagnostic techniques, FMT (Fecal Microbiota Transplantation‐standardization Study Group, 2020), selective microbiota transplantation, spore‐based therapy (Feuerstadt et al., 2022), phage therapy (Federici et al., 2022), interventional techniques for microbiome diagnosis and treatment, conservation innovations for human microbiome diversity (Ke et al., 2022), the basis of diagnosis and treatment for multisystem diseases, healthcare policies and medical education.

Understanding the classification and operational mechanism of microbiota medicine is worth referencing the development history of transfusion medicine. Transfusion medicine has undergone a long and tortuous exploration process, based on the invention of disinfection methods, the development of infusion methods, the application of anticoagulants, the discovery of blood types, the establishment of blood banks and the development of blood component transfusion, before gradually developing into transfusion medicine as a medical technology. Taking China as an example, in 2016, the Chinese government officially approved transfusion medicine as an independent discipline under clinical medicine. One of the core technologies of microbiome therapy is FMT. The material source for FMT and blood transfusion comes from the intestinal and vascular systems of healthy donors, respectively. Both use the isolated and washed components in the laboratory to treat diseases.

## HOW TO DEVELOP MICROBIOTA MEDICINE?

Technology is not equivalent to science, and researchers need to have more concerns about the development of technology than the development of disciplines. Microbiota diagnosis and treatment technology, like other technologies, is prone to technological alienation, which refers to the distorted, deviated and disordered state that occurs during the process of technological development, and becomes a negative force that affects medical progress. Due to the profit‐driven nature of technology, excessive, abusive and even deceptive microbiota diagnostic testing, treatment technology and health products can lead to illegal profiteering and destructive development of technology, eventually resulting in government punishment.

As a clinical medicine discipline, microbiota medicine can attract strategic investment in response to emerging technologies, which can support and buffer the difficulties faced by technology development with disciplinary background. Due to the natural curiosity, more doctors and scientists are joining the field of microbiota medicine, contributing diverse inventions and discoveries to the discipline and promoting the development of technology and medicine.

Therefore, paying attention to the sustainable development of microbiota medicine as a clinical medicine discipline is more powerful than focussing on microbiota diagnosis and treatment technology itself to influence the government to provide more support, activate diversified social capital investment in this field, form a huge basic population, shape a multilevel structure of knowledge creation and thus form a more stable disciplinary ecosystem. The development path of microbiota medicine is essentially the process of forming a sustainable disciplinary. Education and teaching, academic organizations and professional journals are the sustainable carriers and promoters of the development of microbiota medicine, among which education is the most core force.

## CONCLUSION

Microbiota medicine, as a new clinical medicine discipline, applies the theories and techniques of microbiome and medicine to the diagnosis, treatment and prevention of diseases, while maintaining sustainable development. The exploration of microbiota medicine involves scientific questions about the human microbiome and diseases, as well as technical issues related to disease diagnosis and treatment. The great need for disease treatment is the driving force behind the development of microbiota medicine, while education is the core force behind its sustained development. It is time to establish microbiota medicine as a new branch of modern clinical medicine and to improve its hierarchical framework in accordance with the nature of medicine, in order to systematically develop its theories, techniques and education. The government and universities should prioritize the development of microbiota medicine, which will contribute to the sustainable development of modern medicine and fulfil the scientific actions required by the United Nations' sustainable development goals.

## AUTHOR CONTRIBUTIONS

All authors participated in this discussion of keynote dialogue in 2023 China Gut Conference. Faming Zhang drafted the manuscript. Weihong Wang, Yongzhan Nie, Jingnan Li and Xingxiang He reviewed the manuscript. All authors read and approved the final paper.

## FUNDING INFORMATION

This project is supported by the National Key Research and Development Program (2021YFA0717004).

## CONFLICT OF INTEREST STATEMENT

Faming Zhang conceived the concept of GenFMTer and transendscopic enteral tubing and related devices. Other authors declare no conflict of interest regarding the content of this editorial.
